# Speciation Studies of Diorganotin(IV) Complexes with 3,3-Bis(1-methylimidazol-2-yl)propionate—Displacement Reaction by DNA Constituents

**DOI:** 10.1155/2013/106357

**Published:** 2013-12-09

**Authors:** Mohamed M. Shoukry, Safaa S. Hassan

**Affiliations:** ^1^Department of Chemistry, Faculty of Science, Islamic University, Madina 170, Saudi Arabia; ^2^Depatment of Chemistry, Faculty of Science, Cairo University, Cairo 12613, Egypt

## Abstract

The interaction of 3,3-bis(1-methylimidazol-2-yl)propionate (BIMP) with dimethyltin(IV) dichloride (DMT), dibutyltin(IV) dichloride (DBT), and diphenyltin(IV) dichloride (DPT) is investigated at 25°C and 0.1 M ionic strength in water for dimethyltin(IV), and in a 50% dioxane-water mixture for dibutyltin(IV) and diphenyltin(IV). The stepwise formation constants of the 1 : 1 and 1 : 2 complexes formed in solution are calculated from potentiometric measurements using the nonlinear least-square program MINIQUAD-75. The concentration distribution of the various complex species is evaluated as a function of pH. Displacement reactions of the coordinated 3,3-bis(1-methylimidazol-2-yl)propionate by inosine and inosine-5′-monophosphate are investigated from calculations based upon equilibrium properties.

## 1. Introduction

There has been tremendous research in recent years concerning the design of nonplatinum chemotherapeutics with the aim to optimize the features of classical platinum drugs constituting the basic cisplatin framework without some of their drawbacks, namely, toxic side effects, inherent intrinsic resistance, and high cost [1]. Among other noteworthy possibilities, some organotin compounds have emerged as a promising class of cancer chemotherapeutics.

The antitumour properties of tin complexes have been established since 1929 [2] and Gielen [3] has published a series of research papers on this subject during the past two decades. The diorganotin (IV) antitumour complexes showed high *in vitro* activity against P388 leukaemia in mice as well as in some human tumour cell lines [4–10]. Numerous diorganotin (IV) derivatives have been found to exhibit high *in vivo* cytotoxicity against P388 lymphocytic leukaemia and to exhibit less or no activity against other murine systems [11]. However, the new *in vitro* human tumour cell screening tests have once again demonstrated the potential of organotin complexes, some of which have exhibited high activity [12], and thus interest in them has been revitalized.

Organotin (IV) compounds exhibiting potent anticancer activity may act via different mechanisms at the molecular level. The binding property of organotin compounds towards DNA, the ultimate drug target molecule, depends essentially on the coordination number/stereochemistry and the nature of groups directly attached to the tin scaffold [13]. Recently, there have been reports of the interaction of Sn compounds with DNA constituents [14–16]. The antitumour activity of the coordination compounds R_2_SnX_2_L_2_ is controlled by the nature of R, leaving groups (X) and the ligand (L). The coordinated ligand (L) favours in some way the transport of the drugs into cells, while the antitumour activity would be exerted by the diorganotin(IV) moiety dissociated from the complex [17]. The latter would interact with nucleic acids, in a similar way as in the case of the widely used anticancer drug cisplatin. Therefore, there is a relationship between the stability of the organotin(IV) compounds and their antitumor activity. In conjunction with our previous studies on organotin (IV) complexes [18–22], the present paper aims to study dimethyl-, dibutyl-, and diphenyltin(IV) complexes with 3,3-bis(1-methylimidazol-2-yl)propionate (BIMP). The displacement reaction of coordinated (BIMP) by inosine and inosine-5′-monophosphate, taken as representative examples of DNA constituents, is investigated. The equilibrium constant for the displacement reaction is a parameter that may be significant to the antitumor activity of organotin(IV) compounds.

## 2. Experimental

**Scheme 1:**
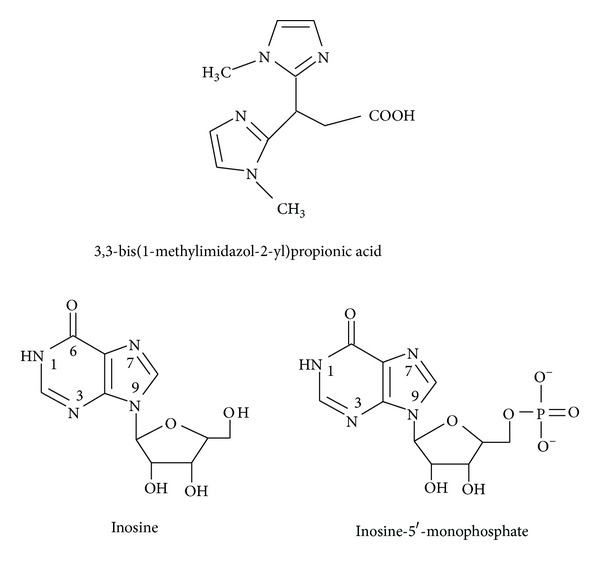
Chemical structure of investigated ligands.

Dimethyltin(IV) dichloride(DMT), dibutyltin(IV) dichloride (DBT), and diphenyltin(IV) dichloride (DPT) were obtained from the Merck Chem. Co. 3,3-bis(1-methylimidazol-2-yl)propionate (BIMP) was prepared as described previously [23]. Inosine and inosine-5'-monophosphate were obtained from Aldrich Chem. Co. The chemical structure of the investigated ligands were given in Scheme 1. Carbonate-free NaOH (titrant) was prepared and standardized against potassium hydrogen phthalate solution. DMT solution was prepared in water, but DBT and DPT solutions were prepared in dioxane. BIMP solution was prepared in the protonated form by dissolving in HNO_3_ solution.

Potentiometric measurements were made using a Metrohm 686 titroprocessor equipped with a 665 Dosimat (Switzerland). The titroprocessor and electrode were calibrated with standard buffer solutions and prepared according to NBS specifications [24]. The titrations were carried out in a purified N_2_ atmosphere using a titration vessel described previously [25]. The temperature was maintained constant by a Colora ultrathermostat. The protonation constants of 3,3-bis(1-methylimidazol-2-yl)propionic acid in the protonated form were determined by titrating 40 mL of a 2.5 × 10^−3 ^M solution. The hydrolysis constants of the DMT, DBT, and DPT compounds were determined by titrating 40 mL solution of concentration 2.5 × 10^−3^ M of each compound. The formation constants of organotin(IV) complexes were determined by titrating 40 mL of solution containing 3,3-bis(1-methylimidazol-2-yl)propionate (2.5 × 10^−3^ M) and a given organotin(IV) compound with a concentration of 1.25 × 10^−3^ M. The titration was performed at 25°C and in water for DMT but in 50% dioxane-water solution for DBT and DPT. The *pK*_w_ in dioxane-water solution was determined as described previously [22, 26]. For this purpose, various amounts of standard NaOH solution were added to a solution containing 0.1 M NaNO_3_. The [OH^−^] was calculated from the amount of base added. The [H^+^] was calculated from the pH value. The product of [OH^−^] and [H^+^] was taken. The mean value obtained in this way for the log concentration product is *pK*_w_ = 15.46 for 50% dioxane-water solution. The equilibrium constants were evaluated from titration data defined by

(1) $$\begin{array}{c} p\left(\text{M}\right)+q\left(\text{L}\right)+r\left(\text{H}\right)⇌{\left(\text{M}\right)}_{p}{\left(\text{L}\right)}_{q}{\left(\text{H}\right)}_{r}, \end{array}$$

(2) $$\begin{array}{c} \beta_{pqr}=\frac{\left[{\left(\text{M}\right)}_{p}{\left(\text{L}\right)}_{q}{\left(\text{H}\right)}_{r}\right]}{{\left[\text{M}\right]}^{p}{\left[\text{L}\right]}^{q}{\left[\text{H}\right]}^{r}}, \end{array}$$

where M, L, and H represent organotin(IV), 3,3-bis(1-methylimidazol-2-yl)propionate, and proton, respectively. The calculations were performed using the computer program MINIQUAD-75 [27]. The stoichiometries and stability constants of the complexes formed were determined by trying various possible composition models. The model selected gave the best statistical fit and was chemically consistent with the titration data without giving any systematic drifts in the magnitudes of various residuals, as described elsewhere [27].  Table 1 lists the equilibrium constants together with their standard deviations as obtained from the program MINIQUAD-75. The concentration distribution diagrams were obtained using the program SPECIES [28].

## 3. Results and Discussion

The protonation constants of 3,3-bis(1-methylimidazol-2-yl) propionic acid were determined by direct potentiometric measurements because all protonation reactions were observed to take place within the potentiometrically measurable pH range. Protonated 3,3-bis(1-methylimidazol-2-yl)propionate behaves as a triprotic acid (H_3_A^2+^), where the differential log protonation constants in aqueous solution were found to be 2.18, 4.20, and 7.11. The first constant is corresponding to the carboxylic group and the second and third constants are corresponding to the protonated imidazole groups.

The hydrolysis of the dimethyltin(IV) cation in aqueous solution was studied by several research groups [29–33]. The potentiometric data were fitted considering the formation of the species with stoichiometric coefficients (1 0 −1), (1 0 −2), (1 0 −3), (1 0 −4), and (2 0 −2) (Table 1). The hydrolysed species may form distannoxanes with a typical 4-membered ring (SnO)_2_ as reported previously [34–36]. This species was not detected, which may be due the poor solubility. The dimerizing ability of the aqua-hydroxo complexes is described by the general equilibrium:

(3) 2(CH3)2Sn(H2O)3(OH)+   (1  0  −1)  ⇌[((CH3)2Sn)2(HO)2(H2O)4]2++2H2O       (2  0  −2).

The dimerization constants can be determined [19] by

(4) log⁡⁡KD=(log⁡⁡β2  0  −2)−2(log⁡⁡β1  0  −1)=(−3.92)−2(−3.39)=2.86.

The potentiometric titration curves of 3,3-bis(1-methylimidazol-2-yl)propionate in the presence and absence of diorganotin(IV) are compared. The complex titration curve is significantly lower than 3,3-bis(1-methylimidazol-2-yl)propionate curve. This corresponds to the formation of a complex species through release of a hydrogen ion.

The complex formation equilibria of dimethyltin(IV) with 3,3-bis(1-methylimidazol-2-yl)propionate is characterized by fitting the potentiometric data to various models. The best model was found to be consistent with the formation of the complexes with stoichiometric coefficients (1 1 0), (1 2 0), and (1 1 −1).

The *pK*_a_ of a coordinated water molecule is calculated [18] by

(5) $$\begin{array}{c} pK_{\text{a}1}=\log\beta_{1\;1\;0}-\log\beta_{1\;1\;-1}. \end{array}$$

The calculated value is 4.77 and higher than that of water molecule coordinated to the free dimethyltin (IV) ion (3.39). This may be due to the elongation of the Sn(IV)-H_2_O bond caused by the coordination of 3,3-bis(1-methylimidazol-2-yl)propionate. This may be explained on the basis that coordination of the ligand will decrease the elecrophilicity of tin, and hence the coordinated water molecule will be weakly bound and consequently will be less acidic.

**Figure 1:**
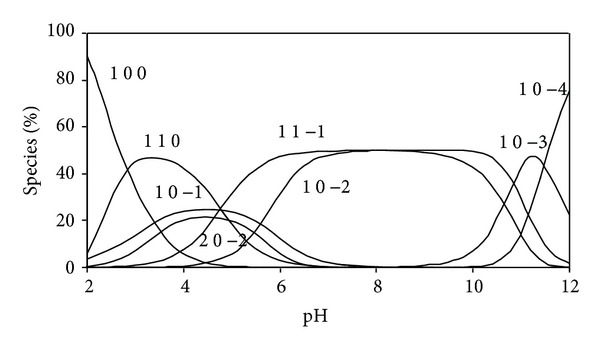
Concentration distribution of various species as a function of pH in the dimethyltin(IV) complexes, (at concentrations of 1.25 mmole/liter for DMT and 2.5 mmole/liter for BIMP) at 25°C.

The concentration distribution diagram (Figure 1) shows that the species (1 1 0) is formed at low pH and predominates at pH 3.3 with an extent of formation of 46%. After pH 4.0, the monohydroxy species (1 1 −1) and hydrolysed species (1 0 −2) start to form and reach the formation percentage of 50% at pH 6.5 for (1 1 −1) species and 49% at pH 7.5 for (1 0 −2) species. The hydrolysed species (1 0 −3) is predominating after pH 10.

The hydrolysis of dibutyltin(IV) and diphenyltin(IV) ions is investigated in 50% dioxane-water solution. The potentiometric data are fitted considering the formation of the species (1 0 −1), (1 0 −2), and (2 0 −1). The monohydroxo-bridged dimer (2 0 −1) is assumed to form through dimerization of the tin(IV) via a hydroxo group, as given in

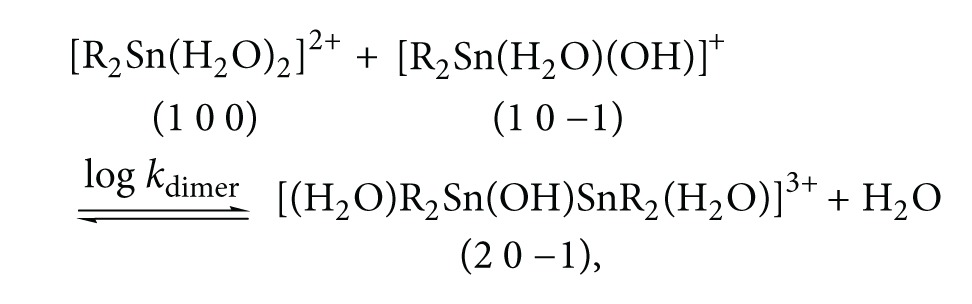
(6)


where R = a butyl- or phenyl-group.

The equilibrium constant for the dimerization reaction (6) is calculated by (7) and amounts to 2.98 for dibutyltin(IV) and 4.89 for diphenyltin(IV):

(7) $$\begin{array}{c} \log K_{\text{dimer}}=\log\beta_{2\;0\;-1}-\log\beta_{1\;0\;-1}. \end{array}$$

The formation of the dihydroxo-bridged dimer (2 0 −2), found for dimethyltin(IV), is not favoured in the case of diphenyltin(IV) and dibutyltin(IV) ions. This may be accounted for on the basis of a steric interaction effect caused by the bulky butyl groups in the case of dibutyltin(IV) and phenyl groups in the case of diphenyltin(IV). This will cause the dimeric form (2 0 −2) to be strained and consequently energetically not favoured.

The *pK*_a_ of coordinated water molecules in dibutyltin(IV) and diphenyltin(IV) species determined in 50% dioxane-water solution are compared. The *pK*_a_ value (−log *β*_1 0 −1_) for diphenyltin(IV) is lower than that of dibutyltin(IV). This may be explained on the premise that the butyl group is having a higher electron donating property. This will increase the electron density on the Sn atom, and consequently the coordinated water will be weakly bound to Sn. This will make the coordinated water less acidic. However, in diphenyltin(IV), the phenyl group is having a higher electron accepting property, which will increase the acidity of the coordinated water molecule.

The potentiometric data for diphenyltin(IV) and dibutyltin(IV) complexes with 3,3-bis(1-methylimidazol-2-yl)propionate are fitted with a model composed of the species (1 1 0) and (1 2 0). The hydrolysed form of the 1 : 1 complex (1 1 −1) is not formed. This may be due to the insolubility of the latter species as a result of its electrical neutrality, and also, due to the hydrophobicity of the phenyl and butyl groups.

**Figure 2:**
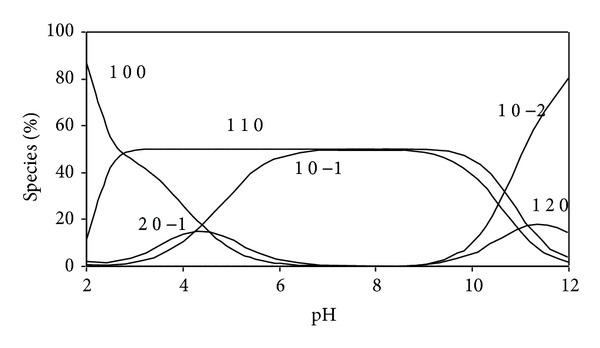
Concentration distribution of various species as a function of pH in the dibutyltin(IV) complexes (at concentrations of 1.25 mmole/liter for DBT and 2.5 mmole/liter for BIMP) at 25°C.

The concentration distribution diagram for dibutyltin(IV) complex, Figure 2, shows that the species (1 1 0) and (2 0 −1) are formed at low pH and reach a formation degree of 50% in the pH range 3–8 for (1 1 0) species and 15% at pH 4.2 for (2 0 −1) species. In the physiological pH range, the predominating species are (1 1 0) and (1 0 −1). The hydrolysed species (1 0 −2) and (1 2 0) species are formed after pH 10. From the biological point of view, it is interesting to note that the (1 1 0) species is predominating in the physiological pH range and consequently the interaction with DNA is feasible.

**Figure 3:**
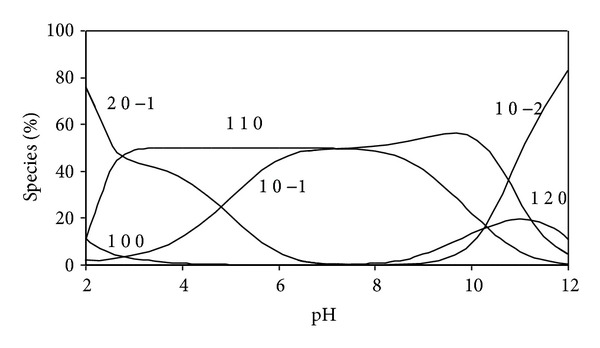
Concentration distribution of various species as a function of pH in the DPT-BIMP system (at concentrations of 1.25 mmole/liter for DPT and 2.5 mmole/liter for BIMP) at 25°C.

The concentration distribution diagram for diphenyltin(IV) complexes, Figure 3, reveals that tin(IV) is hydrolysed giving (2 0 −1) species with a maximum formation degree of 78% at pH 2.0. The complex species (1 1 0) reaches the maximum concentration of 52% in the pH range 2.8–8.0. Therefore, in the physiological pH range, the diphenyltin(IV) complex can interact with DNA constituents.

### 3.1. Displacement Reaction of 3,3-Bis(1-methylimidazol-2-yl)propionate Coordinated to Dimethyltin(IV) by Inosine and IMP

It was reported that DNA constituents [37, 38] have high affinity for dimethyltin(IV), dibutyltin(IV), and diphenyltin(IV), which may have important biological implications since the interaction with DNA is thought to be responsible for the antitumour activity of related complexes [39]. The antitumour activity of a diorganotin-(amine) complex is based on the displacement of the amine by DNA [40]. Consequently, the equilibrium constant for such conversion is of biological significance. Consider inosine as a typical DNA constituent (presented by HB) and 3,3-bis(1-methylimidazol-2-yl)propionate (presented by H_3_A^2+^). The equilibria involved in complex-formation and displacement reactions may be presented as

(8) $$\begin{array}{c} \text{HB}⇌{\text{B}}^{-}+{\text{H}}^{+} \end{array}$$

(9) $$\begin{array}{c} {\text{H}}_{3}{\text{A}}^{2+}⇌{\text{A}}^{-}+{3\text{H}}^{+} \end{array}$$

(10) (CH3)2Sn2++B−⇌(CH3)2Sn(B)+  (1  0  0)        (1  1  0)

(11) $$\begin{array}{c} \beta_{110}{\left({\text{CH}}_{3}\right)}_{2}\text{Sn}{\left(\text{B}\right)}^{+}=\frac{\left[{\left({\text{CH}}_{3}\right)}_{2}\text{Sn}{\left(\text{B}\right)}^{+}\right]}{\left[{\left({\text{CH}}_{3}\right)}_{2}{\text{Sn}}^{2+}\right]\left[{\text{B}}^{-}\right]} \end{array}$$

(12) (CH3)2Sn2++A−⇌(CH3)2Sn(A)+  (1  0  0)        (1  1  0)

(13) $$\begin{array}{c} \beta_{110}{\left({\text{CH}}_{3}\right)}_{2}\text{Sn}{\left(\text{A}\right)}^{+}=\frac{\left[{\left({\text{CH}}_{3}\right)}_{2}\text{Sn}{\left(\text{A}\right)}^{+}\right]}{\left[{\left({\text{CH}}_{3}\right)}_{2}{\text{Sn}}^{2+}\right]\left[{\text{A}}^{-}\right]} \end{array}$$

(14) $$\begin{array}{c} {\left({\text{CH}}_{3}\right)}_{2}\text{Sn}{\left(\text{A}\right)}^{+}+{\text{B}}^{-}\overset{K_{\text{eq}}}{⇌}{\left({\text{CH}}_{3}\right)}_{2}\text{Sn}{\left(\text{B}\right)}^{+}+{\text{A}}^{-}. \end{array}$$

The equilibrium constant for the displacement reaction is given by

(15) $$\begin{array}{c} K_{\text{eq}}=\frac{\left[{\left({\text{CH}}_{3}\right)}_{2}\text{Sn}{\left(\text{B}\right)}^{+}\right]\left[{\text{A}}^{-}\right]}{\left[{\left({\text{CH}}_{3}\right)}_{2}\text{Sn}{\left(\text{A}\right)}^{+}\right]\left[{\text{B}}^{-}\right]}. \end{array}$$

Substitution results in

(16) $$\begin{array}{c} K_{\text{eq}}=\frac{\beta_{110}^{{\left({\text{CH}}_{3}\right)}_{2}\text{Sn}{\left(\text{B}\right)}^{+}}}{\beta_{110}^{{\left({\text{CH}}_{3}\right)}_{2}\text{Sn}{\left(\text{A}\right)}^{+}}}. \end{array}$$

The log *β*_110_ value for dimethyltin(IV) complex with 3,3-bis(1-methylimidazol-2-yl)propionate [(CH_3_)_2_Sn(A)^+^], given in Table 1, amounts to 9.47. The corresponding log *β*_110_ for the inosine complex with the dimethyltin(IV) ions, taken from literature [22], is 8.66. Substitution in (16) results in log *K*_eq_ = −0.81. In the same way, the equilibrium constant for the displacement reaction of coordinated 3,3-bis(1-methylimidazol-2-yl)propionate by inosine-5′-monophosphate was calculated using the formation constant of dimethyltin(IV) complex with inosine-5′-monophosphate, taken from the literature [22], amounting to 11.90. The corresponding log *K*_eq_ value is 2.43. The displacement constant values indicate the ability of inosine and inosine-5′-monophosphate to displace 3,3-bis(1-methylimidazol-2-yl)propionate from its diorganotin (IV) complexes and to what extent the amine complex can interact with the DNA constituent, the main target in tumour chemotherapy. Comparison of the displacement constant values for inosine and inosine-5′-monophosphate showed that the nucleotide IMP has the higher value. This can be explained on the basis of the different columbic force of attraction between the dipositively charged diorganotin(IV) ion and IMP having extra negative charges on the phosphate group. The increase of columbic force of attraction between diorganotin(IV) and IMP will facilitate the release of coordinated 3,3-bis(1-methylimidazol-2-yl)propionate.

## 4. Conclusion

The present investigation describes complex formation equilibria of 3,3-bis(1-methylimidazol-2-yl)propionate with dimethyltin(IV), dibutyltin(IV), and diphenyltin(IV) ions. The results show the formation of 1 : 1 and 1 : 2 complexes. The displacement reaction of 3,3-bis(1-methylimidazol-2-yl)propionate coordinated to dimethyltin(IV) by DNA constituent as inosine and inosine-5′-monophosphate was investigated and a measure of the displacement was calculated. The results reveal to what extent DNA, the major target in tumour therapy, can displace the coordinated amine, 3,3-bis(1-methylimidazol-2-yl)propionate, and interact with dimethyltin(IV). These data are expected to contribute to the chemistry of organotin(IV) compounds as potential antitumor agents.

## Figures and Tables

**Table 1:** Formation constants of organotin(IV) complexes with 3,3-bis(1-methylimidazol-2-yl)propionate in water for DMT and in 50% (v/v) dioxane-water solution for DBT and DPT, at 25°C and 0.1 M ionic strength.

Organotin(IV)	*p* ^a^	*q* ^a^	*r* ^a^	log*β* ^b^
DMT	0	1	1	7.11 (0.01)
	0	1	2	11.31 (0.02)
	0	1	3	13.49 (0.03)
	1	0	−1	−3.39 (0.01)
	1	0	−2	−8.99 (0.01)
	1	0	−3	−19.83 (0.01)
	1	0	−4	−31.30 (0.01)
	2	0	−2	−3.92 (0.01)
	1	1	0	9.47 (0.02)
	1	2	0	14.78 (0.11)
	1	1	−1	4.70 (0.11)

DBT	0	1	1	6.66 (0.01)
	0	1	2	10.98 (0.02)
	0	1	3	14.43 (0.02)
	1	0	−1	−4.39 (0.01)
	1	0	−2	−15.05 (0.02)
	2	0	−1	−1.41 (0.04)
	1	1	0	10.56 (0.06)
	1	2	0	14.72 (0.09)

DPT	0	1	1	6.66 (0.01)
	0	1	2	10.98 (0.02)
	0	1	3	14.43 (0.02)
	1	0	−1	−2.82 (0.10)
	1	0	−2	−13.58 (0.10)
	2	0	−1	2.07 (0.10)
	1	1	0	11.43 (0.03)
	1	2	0	15.87 (0.04)

^a^The *p*, *q*, and *r *are the stoichiometric coefficients corresponding to organotin(IV), 3,3-bis(1-methylimidazol-2-yl)propionate, and H^+^, respectively; ^b^Standard deviations are given in parentheses.
